# Potential of mTOR inhibitors for the treatment of subependymal giant cell astrocytomas in tuberous sclerosis complex

**DOI:** 10.18632/aging.100298

**Published:** 2011-03-12

**Authors:** Philippe Major

**Affiliations:** Department of Pediatrics, Neurology Service, Centre Hospitalier Universitaire Sainte-Justine, Université de Montréal, Montréal, Québec, Canada

**Keywords:** Rapamycin, mTOR, tuberous sclerosis complex, subependymal giant cell astrocytoma

## Abstract

Rapamycin inhibits the mTOR (target of rapamycin) pathway and extends lifespan in multiple species. The tuberous sclerosis complex (TSC) protein is a negative regulator of mTOR. In humans, loss of the TSC protein results in a disorder characterized clinically by the growth of benign tumors in multiple organs, due to overactivation of mTOR inhibition. Subependymal giant cell astrocytomas (SEGAs) are benign brain tumors associated with TSC that have traditionally been treated by surgery, but for which mTOR inhibitors have recently been suggested as potential alternative treatments. The duration of mTOR treatment for SEGAs might have to be prolonged, probably lifelong, because SEGAs usually grow back after treatment is stopped. This cohort of patients who will experience prolonged exposure to mTOR inhibitors should be carefully followed longitudinally to better document long term side effects, but also to compare their longevity with the one of similar patients with TSC. These patients represent a unique opportunity to study the potential anti-aging properties of mTOR inhibitors in humans.

Rapamycin (also called sirolimus) is an immunosuppressive drug that has recently been shown to extend lifespan in multiple species including mammals [1]. This anti-aging property is presumably related to the mTOR (mammalian target of rapamycin) inhibition properties of rapamycin. The mTOR pathway is crucial for the coordination of growth in response to energy status, stress, and nutrient availability [2, 3].

The potential anti-aging properties of rapamycin and of other mTOR inhibitors, such as RAD001 (everolimus), and CCI-779 (temsirolimus) are of great interest. Unfortunately, the side effects related to these drugs preclude the undertaking of research trials about their impacts on aging in healthy individuals. Considering this obstacle, experts in the field of aging have suggested that the potential anti-aging drugs should be introduced to the clinical trials for therapy of particular diseases and then be approved for prevention of all age-related diseases in healthy individuals [4]. In this context, tuberous sclerosis complex (TSC) seems to be an ideal disease model where the potential of mTOR inhibitors can be assessed because these drugs are increasingly being tested and used clinically to treat certain aspects of this condition [5].

TSC is an autosomal dominant disorder caused by the inactivation in one of two tumor suppressor genes, hamartin (TSC1) or tuberin (TSC2). In the normal state, the hamartin-tuberin complex activates the protein Rheb, which inhibits mTOR. If a TSC mutation is present, mTOR is constitutively activated, leading to abnormal cellular proliferation, ribosome biogenesis, and mRNA translation (see [2] for complete review of the mTOR molecular pathway). In consequence, TSC is characterized clinically by the growth of benign tumors in multiple organs, including the brain, the heart, the kidneys, the lungs, and the skin [6]. Its incidence is estimated at 1 in 6000 live births [7]. The severity of the disease is highly variable, ranging from mild skin manifestations to intractable epilepsy, mental retardation, and autism [8].

The only report studying specifically the causes of death in TSC was performed at the Mayo clinic [9]. Overall, the survival curves showed a decreased survival for patients with TSC compared with the general population. Of the 355 patients with TSC followed, 40 died of causes related to TSC, with renal disease being the most common cause of death (11/40). Ten patients died as a consequence of brain tumors and four patients died of lymphangioleiomyomatosis (LAM). Thirteen patients with severe mental impairment passed away due to status epilepticus or bronchopneumonia. One baby died of cardiac failure and one child died of rupture of an aneurysm of the thoracic aorta.

The main current clinical complication related to TSC for which treatment with mTOR inhibitors is indicated are subependymal giant cell astrocytomas (SEGA). This complication affects approximately 15% of patients with TSC and it occurs in the pediatric age group [10]. SEGAs tend to lose their propensity to grow in the early twenties. They are slow-growing benign tumors of mixed glioneuronal lineage that arise from the growth of pre-existing subependymal nodules, which are asymptomatic lesions that protrude from the walls of the ventricles [10]. SEGAs most commonly grow near the foramen of Monro. This can lead to obstruction of the normal cerebrospinal fluid circulation and subsequent intracranial hypertension that can potential be fatal if left untreated. The distinction between a SEGA and a subependymal nodule is still debated. Generally, a clinical diagnosis of SEGA is made when there are symptoms of intracranial hypertension, papilledema, or radiological evidence of hydrocephalus or interval growth.

The traditional management approach is to monitor SEGAs with periodic neuroimaging and to resect those that exhibit growth and/or cause clinical signs of intracranial hypertension. This approach is being challenged by recent observations that suggest that mTOR inhibitors, such as rapamycin (sirolimus) and RAD001 (everolimus), can induce partial regression of SEGAs [11, 12, 13]. The first report showing clear regression of SEGAs in five patients with the use of rapamycin was published in 2006 [11]. Recently, a phase II trial [13] using everolimus to treat SEGAs in 28 patients with TSC showed SEGA reduction of at least 30% in 21 patients (75%) and at least 50% in 9 patients (32%). Everolimus was well tolerated as only single cases of grade 3 treatment-related sinusitis, pneumonia, viral bronchitis, tooth infection, stomatitis, and leukopenia were reported.

These observations suggest that mTOR inhibitors could serve as an acceptable alternative treatment to SEGA surgery. Renal angiomyolipomas and lymphangioleimyomatosis (LAM) are other TSC manifestations for which mTOR inhibitors have proven potential efficacy [14]. In addition, animal models of TSC have suggested that mTOR inhibitors could have beneficial effects on cognitive deficits [15] and on epileptogenesis [16]. Whether similar benefits would be observed in humans with TSC is still unknown. Research trials are ongoing and should soon provide answers to these questions.

Other important questions remain regarding the use of mTOR inhibitors for the treatment of SEGA in TSC. Side effects, especially long term side effects, and optimal duration of treatment are still under investigation. The short-term side effects related to rapamycin are generally considered acceptable. The most common side effects are oral ulcers, acneiform rash, thrombocytopenia, hyperlipidemia, impaired wound healing, and immunosuppression [14]. Long term side effects are less known. For example, reports from the literature related to the use of rapamycin for kidney transplant prevention suggested that rapamycin might be associated with impaired spermatogenesis and, as a corollary, may reduce male fertility [17]. This observation might not be applicable to other patients populations, but requires further investigation.

The duration of treatment might be prolonged, probably lifelong. There is clear evidence that SEGAs grow back after the mTOR inhibitor is stopped [11]. Most experts currently recommend continuation of mTOR inhibitors at the lowest efficacious dose. This cohort of patients who will experience prolonged exposure to mTOR inhibitors should be carefully followed longitudinally to better document long term side effects, but also to compare their longevity with the one of similar patients with TSC. These patients represent a unique opportunity to study the potential anti-aging properties of mTOR inhibitors in humans.

In conclusion, a new treatment era has begun in the field of TSC since the discovery of the potential beneficial effects of mTOR inhibitors. Although the use of mTOR inhibitors is becoming increasingly accepted, especially for the treatment of SEGAs in TSC, questions now remain about the duration of treatment and long term side effects. Whether mTOR inhibitors will have a significant impact on longevity in TSC is unknown, but warrants attention as mTOR inhibitors are increasingly recognized as anti-aging drugs in animal models. Long-term prospective studies in patients with TSC might provide evidence about the potential anti-aging properties of mTOR inhibitors in humans.
